# Enzymatic Laser‐Induced Graphene Biosensor for Electrochemical Sensing of the Herbicide Glyphosate

**DOI:** 10.1002/gch2.202200057

**Published:** 2022-07-26

**Authors:** Zachary T. Johnson, Nathan Jared, John K. Peterson, Jingzhe Li, Emily A. Smith, Scott A. Walper, Shelby L. Hooe, Joyce C. Breger, Igor L. Medintz, Carmen Gomes, Jonathan C. Claussen

**Affiliations:** ^1^ Department of Mechanical Engineering Iowa State University Ames IA 50011 USA; ^2^ Department of Chemistry Iowa State University Ames IA 50011 USA; ^3^ The Ames Laboratory U.S. Department of Energy Ames IA 50011 USA; ^4^ Center for Bio/Molecular Science and Engineering, Code 6900 U.S. Naval Research Laboratory Washington, D.C 20375 USA; ^5^ National Research Council Washington, DC 20001 USA

**Keywords:** biosensors, glycine oxidase, glyphosate, herbicides, laser‐induced graphene

## Abstract

Glyphosate is a globally applied herbicide yet it has been relatively undetectable in‐field samples outside of gold‐standard techniques. Its presumed nontoxicity toward humans has been contested by the International Agency for Research on Cancer, while it has been detected in farmers’ urine, surface waters and crop residues. Rapid, on‐site detection of glyphosate is hindered by lack of field‐deployable and easy‐to‐use sensors that circumvent sample transportation to limited laboratories that possess the equipment needed for detection. Herein, the flavoenzyme, glycine oxidase, immobilized on platinum‐decorated laser‐induced graphene (LIG) is used for selective detection of glyphosate as it is a substrate for GlyOx. The LIG platform provides a scaffold for enzyme attachment while maintaining the electronic and surface properties of graphene. The sensor exhibits a linear range of 10–260 **µ** m, detection limit of 3.03 **µ** m, and sensitivity of 0.991 nA **µ** m ^−1^. The sensor shows minimal interference from the commonly used herbicides and insecticides: atrazine, 2,4‐dichlorophenoxyacetic acid, dicamba, parathion‐methyl, paraoxon‐methyl, malathion, chlorpyrifos, thiamethoxam, clothianidin, and imidacloprid. Sensor function is further tested in complex river water and crop residue fluids, which validate this platform as a scalable, direct‐write, and selective method of glyphosate detection for herbicide mapping and food analysis.

## Graphical abstract

A platinum‐decorated, glycine oxidase (GlyOx) functionalized, laser‐induced graphene (LIG) sensor selectively detects the broad range herbicide glyphosate against common herbicides and insecticides. The sensor displays a linear sensing range of 10–260 µm, limit of detection (LOD) of 3.03 µm, and response time of 150 s. Furthermore, the sensor detects glyphosate in complex fluids.

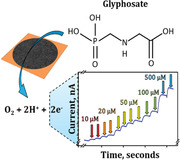

## 1 Introduction

Glyphosate, N‐(phosphonomethyl) glycine, has grown as a successful agrochemical since its creation in the 1970's due to its efficiency in killing weeds, enabling of no‐till cropping, and synchronization with the adoption of genetically modified crops that possess glyphosate resistance.^[^ ^1^ ^]^ Glyphosate maintains the largest share of herbicide use with an application of 113.4 million kg in the United States and 747 million kg globally, as reported in 2014.^[^ ^2^ ^]^ Although glyphosate is largely believed to be non‐toxic to animals and humans, its accumulation in ground water after heavy rains and movement into surface waters has increased concern about its larger environmental and human health impact.^[^ ^3^ ^]^ Notably, glyphosate has been detected in concentrations up to 27.8 µg L^−1^ in 44% of Midwestern stream samples from a study performed during the 2013 Midwestern growing season.^[^ ^4^ ^]^ A South Carolina and Minnesota study showed positive glyphosate detection in farmers’ urine during their application cycle of up to 3.2 µg L^−1^,^[^ ^5^ ^]^ while a similar study in Wisconsin confirmed a maximum sample concentration of 12 µg kg^−1^.^[^ ^6^ ^]^ Perhaps more worrisome, the International Agency for Research on Cancer (IARC) classified glyphosate as a “probable human carcinogen,” a claim highly contested by the Environmental Protection Agency (EPA) and European Food Safety Authority (EFSA),^[^ ^7^ ^]^ though critiques have been made regarding both the EPA's and European Food Safety Authority's analysis methods^[^ ^8^, ^9^ ^]^ as higher occupational exposures and hazard‐based assessments were not considered.^[^ ^5^, ^10^ ^]^ Additionally, multiple studies have tied chronic glyphosate exposure to various health hazards, some of which include heart disease,^[^ ^11^ ^]^ non‐Hodgkin lymphoma,^[^ ^12^ ^]^ Parkinson's disease^[^ ^13^ ^]^ and pregnancy issues.^[^ ^14^ ^]^ However, due to poor pesticide exposure assessment, the role of low‐level but frequent pesticide exposure from environmental residues cannot be conclusively pinpointed as the underlying cause of disease.^[^ ^15^ ^]^ Knowledge of pesticide drift, runoff, exposure, and endpoints would also provide critically important information to help oversee appropriate pesticide stewardship and application methodologies that keep pesticides in target areas or spaces at appropriate times to reduce environmental contamination. Hence, wide‐scale deployment of in‐field sensors that are much more cost effective than shipping samples back to a laboratory are needed to effectively quantify, track and map glyphosate contamination in watersheds, drinking water, food samples and agricultural fields. Rapid in‐field testing near the point of use is especially important for glyphosate due to its water solubility, low volatility and favored complexing behavior, which can significantly dampen efforts to quantify contamination.^[^ ^12^ ^]^ Current methods to detect glyphosate include laboratory techniques (e.g., ELISA, liquid/gas chromatography, mass spectroscopy) that often require expensive equipment, complex protocols, derivatization steps, sample pretreatment, and sample transportation to the test laboratory.^[^ ^12^, ^16^, ^17^, ^18^ ^]^ A commercially available, field‐deployable glyphosate sensor does not currently exist.

There are, however, a number of sensing modalities to monitor glyphosate beyond typical laboratory techniques including using chemiluminescence^[^ ^19^ ^]^ and field‐effect transistors (FETs)^[^ ^20^ ^]^ in conjunction with nano‐zinc oxide decorated carbon nanotubes and carboxylate‐functionalized polythiophene as glyphosate reactive networks, respectively. These sensors have achieved impressively low limits of detection (0.8 pg L^−1^ and 0.38 fm, respectively). However, such devices require intensive cleanroom manufacturing steps that increase biosensor cost/complexity and hence render them not suitable for in‐field environmental monitoring.^[^ ^21^, ^22^, ^23^ ^]^ Moreover, for FET‐based sensors, rigorous studies of interferents were not performed against potential organophosphates and other common herbicides, so the specificity of this sensor design remains unclear. Chemiluminescent methodologies often include complex equipment for detection that are not field‐deployable and are challenging to operate in turbid field samples; such sensors are generally not appropriate for in‐field glyphosate sensing.

Electrochemical sensing of glyphosate could provide a low‐cost, field deployable method to monitor and map large areas of glyphosate contamination in the field. Such sensors provide a digital readout of the concentration of the target marker even within turbid field samples without the need to pre‐label samples as is the case with lateral flow assays. There are a wide variety of electrochemical sensors that utilize distinct biorecognition agents including molecularly imprinted polymers (MIPs),^[^ ^24^, ^25^ ^]^ anti‐glyphosate antibodies^[^ ^26^, ^27^ ^]^ and enzymes, including horseradish peroxidase^[^ ^28^ ^]^ and acetylcholinesterase^[^ ^29^ ^]^ to sense glyphosate. In the case of the molecularly imprinted polymers (MIPs), the sensors were tested against multiple interferents, validated in complex media, and possess low limits of detection, but repeat responses were noted to decline in successive tests as sensor regeneration could only occur once.^[^ ^24^ ^]^ Glyphosate immunosensors demonstrated remarkable limits of detection but relied upon the change in signal attributed to antibody–antigen binding and render the sensors useless after one test. Acetylcholinesterase and horseradish peroxidase sensors possess challenges regarding specificity as well since research indicates both are easily inhibited by other pesticides (e.g., atrazine, parathion, malathion, or paraoxon).^[^ ^30^, ^31^, ^32^, ^33^, ^34^, ^35^ ^]^ Moreover, these examples of electrochemical glyphosate sensors utilize gold, copper and indium tin oxide‐based electrodes that further increase the cost of the sensors, may demand specific surface attachment requirements, and do not provide a high surface area environment for increased biorecognition loading and heterogenous charge transport.

We contend that carbon‐based materials exhibit promising electrical properties, electrocatalytic sites, large specific surface area/porosity, and low cost that is well‐suited for in‐field environmental sensing.^[^ ^36^, ^37^, ^38^ ^]^ Researchers have detected glyphosate fluorescently with carbon and graphene dots,^[^ ^39^, ^40^, ^41^, ^42^ ^]^ electrochemically with carbon paste and screen printed electrodes functionalized with reduced graphene oxide,^[^ ^43^, ^44^, ^45^, ^46^, ^47^ ^]^ and optically with a graphene/zinc oxide nanocomposite.^[^ ^48^ ^]^ However, carbon dot synthesis typically requires various toxic chemical reagents as well as high thermal or electrical energy input;^[^ ^49^ ^]^ graphene synthesis usually involves mechanical/chemical exfoliation, thermal decomposition of silicon carbide or chemical vapor deposition,^[^ ^50^ ^]^ while screen, aerosol, gravure and inkjet printed graphene require ink formulation and post‐print annealing, masks or stencils, and aggressive solvents like *N*‐methyl‐2‐pyrrolidone (NMP) and *N*,*N*‐dimethyl formamide (DMF) or even low boiling point solvents that suffer from poor graphene dispersion.^[^ ^51^ ^]^ Laser‐induced graphene (LIG), a direct‐write, laser engraving process, circumvents the need for tedious graphene synthesis techniques as well as the need to create solution‐phase inks, print, and perform post‐print annealing.^[^ ^52^, ^53^, ^54^ ^]^ With LIG, electrochemical graphene‐based electrodes can be synthesized, patterned and annealed all with a single CO_2_ laser pass, which converts sp^3^‐carbon atoms into sp^2^‐carbon atoms by reaching local temperatures of 1000 °C, effectively graphitizing the polymer into a conductive graphene platform.^[^ ^53^, ^55^ ^]^ The laser processing can be tuned in order to introduce functional groups that are conducive to enzyme and antibody crosslinking.^[^ ^56^ ^]^ Furthermore, researchers have developed a plethora of LIG devices for electroacoustics,^[^ ^57^ ^]^ *Salmonella* ^[^ ^58^ ^]^ and SARS‐CoV‐2^[^ ^59^ ^]^ electrochemical immunosensing, electrochemical sweat^[^ ^60^, ^61^, ^62^ ^]^ and soil nutrient monitoring,^[^ ^63^ ^]^ and thermally conductive embeddable gas detection.^[^ ^64^ ^]^ With regard to pesticides, LIG has been used to electrochemically detect neonicotinoids^[^ ^65^ ^]^ and has been coupled with horseradish peroxidase for the detection of atrazine^[^ ^34^ ^]^ as well as organophosphorus hydrolase^[^ ^66^ ^]^ and acetylcholinesterase^[^ ^67^ ^]^ for the detection of methyl parathion. These reports demonstrated the application of LIG as a viable pesticide sensor and biorecognition agent scaffold. However, there does not currently exist in literature an electrochemical sensor that incorporates the enzyme glycine oxidase much less uses an LIG electrode for the selective detection of glyphosate.

Herein, we introduce the concept of using LIG for the detection of glyphosate. The LIG circuit is decorated with platinum (Pt) nanoparticles to further improve its electrochemical reactivity and is further biofunctionalized with glycine oxidase (GlyOx) to permit selective monitoring of glyphosate. The resultant Pt‐GlyOx‐LIG sensor demonstrated a glyphosate linear sensing range of 10–260 µm, response time of 150 s, sensitivity of 0.991 nA µm ^−1^, and a limit of detection (LOD) of 3.03 µm. The sensor exhibited minimal interference from a variety of commonly used herbicides, organophosphates, and neonicotinoids. To prove complex fluid validation for future field deployment, the sensor was tested in spiked corn and soybean residues as well as river water samples from the South Skunk River in Iowa. This low‐cost, LIG‐based senor could be deployed on a large scale and consequently be used to monitor and map large agricultural watersheds.

## 2 Results and Discussion

### 2.1 Pt‐LIG Electrode Characterization

**Figure 1:**
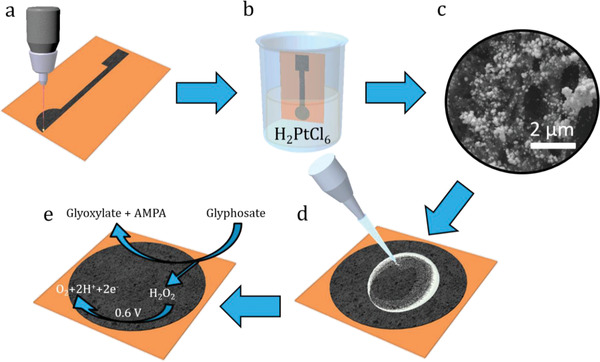
Fabrication schematic of the glyphosate sensor and mechanism of detection: a) CO_2_ laser conversion of polyimide into laser‐induced graphene (LIG); b) platinum (Pt) electrodeposition at −0.5 V versus Ag/AgCl; c) SEM of Pt‐decorated LIG at 15 000× magnification; d) drop coat of glutaraldehyde, flavin adenine dinucleotide and glycine oxidase; and e) simplified mechanism of glyphosate detection through the electrochemical oxidation of hydrogen peroxide at 0.6 V versus Ag/AgCl.

**Figure 2:**
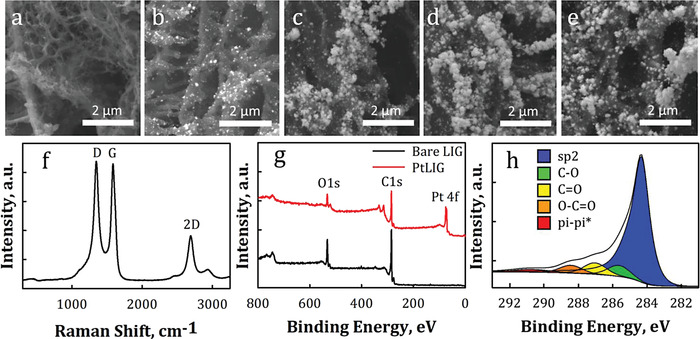
Materials characterization of platinum‐decorated LIG: SEM images at 15 000× magnification of a) bare LIG, b) 1 pulse of Pt, c) 2 pulses of Pt, d) 3 pulses of Pt, e) 4 pulses of Pt, f) Raman spectrum, g) XPS, and h) deconvoluted XPS of C 1s.

A full description of the materials and methods utilized is found below. The LIG electrode fabrication process and sensing modality are shown in **Figure** **1**. To create the sensors, polyimide film was first laser irradiated and converted into LIG through the use of a CO_2_ laser. Platinum nanoparticles (PtNPs) were next electrodeposited onto the LIG through a 30 s step function pulse at −0.5 V versus Ag/AgCl. The reduction of the Pt solution allowed the PtNPs to deposit onto LIG defects (Figure 1c). LIG and PtNP materials characterization were performed using scanning electron microscopy (SEM) and X‐ray photoelectron spectroscopy (XPS) to elucidate the material properties of the electrodes and verify the quality of the fabrication steps (**Figure** **2**). SEMs were acquired at varying Pt  pulses to visualize the LIG morphology and the increase in Pt coverage of LIG defects (Figure 2a–e). The LIG pore, Pt clusters, and PtNPs dimensions varied between 1–3, 81–157 and 33–43 nm, respectively, while the LIG cross‐sectional thickness ranged between 32–37 µm (Figure S1, Supporting Information). The Raman spectrum (Figure 2f) of bare LIG displayed G, D, and 2D peaks that are characteristic of graphene materials. For the bare LIG, the intensity ratio of the 2D to G peak, denoted as *I*_2D_/*I*_G_, was 0.38 ± 0.04, which is indicative of multilayer graphene.^[^ ^68^ ^]^ More specifically, the G peak is a measurement of first‐order scattering of the E_2_g mode, which represents the graphitic nature of the material^[^ ^69^ ^]^ and was observed at 1588 cm^−1^. The D peak correlates to lattice defects^[^ ^70^ ^]^ and was observed at 1347 cm^−1^. The 2D peak represents the second order of zone‐boundary phonons,^[^ ^71^ ^]^ indicative of the graphene structure,^[^ ^68^ ^]^ and was observed at 2691 cm^−1^. Material characterization analysis through XPS indicates the composition of the carbon (C), oxygen (O), and Pt in the PtLIG samples. XPS (Figure 2g) displayed strong C 1s and O 1s peaks near 284.8 and 532.8 eV, respectively. The deconvoluted C 1s data (Figure 2h) showed various carbon bonds including sp2 hybridized carbons C=C/C—C (284.6 eV), hydroxyls C—O/C—OH (286.6 eV), carbonyls C=O (287.9 eV), and carboxyls C(=O)—OH (289.2 eV). The C/O ratio of bare LIG was 5.1 and confirmed the effective carbonization of the polyimide film into graphene. Similarly, C 1s and O 1s peaks were found in the Pt‐decorated samples and showed a peak in the Pt 4f region near 74.8 eV. The Pt 4f region confirmed the presence of metallic Pt and PtO_2_ at 71.1 and 74.5 eV, respectively (Figure S2, Supporting Information), and is supported by similar Pt characterization studies.^[^ ^72^ ^]^

**Figure 3:**
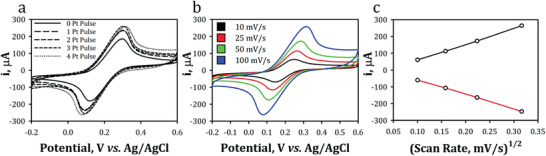
Electrochemical characterization of laser‐induced graphene (LIG) electrode: a) cyclic voltammograms (CV) in 5 mm ferri/ferrocyanide of increasing Pt pulses, b) CV in 5 mm ferri/ferrocyanide of a 4 × Pt pulse electrode at increasing scan rates, and c) Randles–Sevcik plot.

The Pt‐LIG electrical and electrochemical properties were next analyzed using sheet resistance and electroactive surface area (ESA) measurements. Bare LIG and Pt‐decorated LIG samples were compared and exhibited sheet resistance values of 20.3 and 19.0 Ω/square, respectively. The Pt‐decorated samples incorporated 4 successive potential pulses to deposit PtNPs on the surface of the LIG. Furthermore, there was an increase in the peak current of cyclic voltammograms (CV) taken at 100 mV s^−1^ at varying Pt deposition pulses (**Figure** **3**a). The increase in peak current correlates to an increase in charge transfer at the electrode surface. This increase can be attributed to increased ESA through successive Pt pulses and is confirmed in Table S1, Supporting Information. ESA was estimated using the Randles–Sevcik equation (Equation (1)) by comparing the anodic/cathodic current peaks against the square root of the CV scan rates (Figure 3b,c). The Randles–Sevcik equation relates the anodic/cathodic peak current to the following: ESA (*A*), redox probe diffusion coefficient (*D*), number of electrons transferred in the redox probe (*n*), scan rate (ν), and the bulk concentration of the redox species (*C*).^[^ ^73^ ^]^ See the Supporting Information for values and calculations.

(1) $$\begin{array}{c} i_{p}=2.69\times 10^{5}AD^{\frac{1}{2}}\;n^{\frac{3}{2}}\;v^{\frac{1}{2}}\;C \end{array}$$

The geometric surface area for a 3 mm diameter electrode is 0.071 cm^2^. The ESA for bare LIG and 4 × Pt‐LIG were calculated as 0.127 and 0.193 cm^2^ and constitutes 179% and 272% of the geometric surface area, respectively. The observed increase in ESA likely occurs due to the amorphous, porous, and 3D nature of the LIG and PtNP structures (Figure 2a–e), which allows the redox probe greater accessibility to edge sites on the LIG.^[^ ^74^, ^75^ ^]^ The deposition of PtNPs and increase in ESA is consistent with similar studies,^[^ ^76^ ^]^ which attributed the increase in ESA to the catalytic activity of the PtNPs. Furthermore, the Randles–Sevcik plot (Figure 3c) shows that increases in the anodic/cathodic peak current behave linearly with the square route of the scan rate, which is an indicator of electrochemical reversibility.^[^ ^77^ ^]^ However, as the scan rate increased, the peaks drifted apart and the peak‐to‐peak separation (Δ*E*_p_) increased, which is representative of a more quasi‐reversible or irreversible system.^[^ ^78^ ^]^ Electrochemical reversibility is determined by the mass transport (*m*_transport_) to the electrode surface and the standard electron rate constant (*k* ^0^), which is synonymous with the heterogenous electron transfer rate (HET). To evaluate *k* ^0^, the transfer coefficient, α, was determined using the Tafel slope method as discussed in Equation S1, Supporting Information.^[^ ^79^ ^]^ The transfer coefficient was calculated as 0.42 ± 0.07 and is an indicator of the symmetry of the redox reaction where a value of 0.5 indicates symmetry about the energy barrier regarding the redox reaction. For the 4 × Pt pulse LIG sample, Δ*E*_p_ ranged between 104 to 241 mV for 10 to 100 mV s^−1^, respectively. The *k* ^0^ was estimated using Δ*E*_p_ and both the Nicholson and Kochi methods^[^ ^79^ ^]^ as expressed in Equations S2–S4, Supporting Information. Rates were determined to be 0.0010 ± 0.0005 and 0.0016 ± 0.0006 cm s^−1^ for the Nicholson and Kochi methods, respectively, which indicated a quasi‐reversible electrochemical system that can be attributed to LIG defects. The criteria for determining reversibility are shown in Tables S2 and S3, Supporting Information, as reported by Aristov.^[^ ^78^ ^]^

### 2.2 Glycine Oxidase and Glyphosate Kinetics

**Figure 4:**
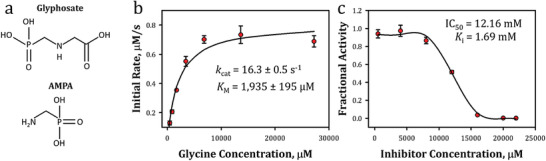
Chemical structures and determination of glycine oxidase Michaelis–Menten and inhibition by glyphosate: a) chemical structures of glyphosate and AMPA, b) Michaelis–Menten plot, and c) fractional activity versus increasing inhibitor concentrations of glyphosate.

**Scheme 1:**
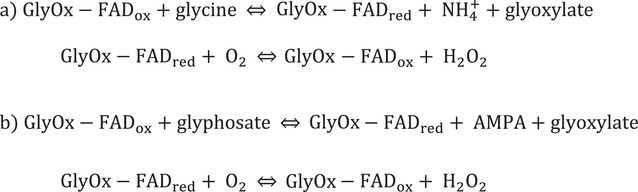
Reaction mechanism of glycine and glyphosate oxidation.^[^ ^82^ ^]^

The biochemical mechanisms behind glycine oxidase catalysis of glyphosate are discussed to help elucidate the workings of the developed sensor. This reaction mechanism takes place within the enzyme's active site, which is located in a cavity that invaginates toward the center of the enzyme. At the bottom of the cavity, the FAD cofactor binding site is located. In the active site, arginine (Arg)^302^ and tyrosine (Tyr)^246^ become hydrogen bonded with one of the two oxygens located on the alpha carboxylate group of glyphosate. The second oxygen of the alpha carboxylate group is then hydrogen bonded with N‐5 of the FAD cofactor. Histidine (His)^244^ uses its carbonyl and Arg^329^ utilizes a side chain nitrogen to then form hydrogen bonds with the alpha carboxylate moiety of glyphosate to stabilize the substrate within the active site.^[^ ^80^ ^]^ Once in the active site, the FAD is able to then extract a hydrogen from the alpha carbon of glyphosate. This then forms an acetate intermediate with an imine group. This falls under nucleophilic attack by a water molecule causing the electrons within the Pi bonds connecting the alpha carbon to the imine group of the acetate intermediate to be shifted to the nitrogen, which then extracts a proton from the bound water molecule forming a hydroxyl group on the alpha carbon. The hydroxyl group then forms a double bond with one of the oxygen's lone pairs forming an unstable carbonyl cation. The formation of this carbonyl causes the bond with the aminomethylphosphonic acid (AMPA) to be broken. AMPA consists of the nitrogen, carbon, and phosphonate group that make up glyphosate. Once broken, AMPA and a cation intermediate of glyoxylate are formed. The primary nitrogen of the AMPA molecule extracts the proton of the carbonyl cation forming glyoxylate as the final product.^[^ ^81^ ^]^ Glyphosate and AMPA chemical structures are shown in **Figure** **4**a. Additionally, the glycine oxidase reaction mechanism for the native substrate, glycine, as well as for glyphosate are shown in **Scheme 1**. ^[^ ^82^ ^]^

Glycine oxidase assays with native glycine substrate were performed to determine the baseline kinetic rates for the enzyme by assaying it against increasing concentrations of glycine. Figure 4b shows representative results from this analysis where the initial rate of 50 nm glycine oxidase is plotted against increasing concentrations of glycine. Analysis of this data within the Michaelis–Menten framework yielded a maximum velocity (*V*_max_) of 0.81 ± 0.02 µm s^−1^, a value of 1935 ± 195 µm for the Michaelis constant (*K*_M_) which reflects substrate affinity, and a catalytic rate (*k*_cat_) of 16.3 ± 0.5 s^−1^. Enzymatic efficiency was estimated by the second order rate constant *k*_cat_/*K*_M_ as 8.4 ± 0.9 mm ^−1^ s^−1^, which outperforms other reported glycine oxidase efficiencies of 0.1, 1.5, 1.3, and 0.9 mm ^−1^ s^−1^; this presumably arises due to both sequence differences along with use of different assay formats, buffers, etc.^[^ ^81^, ^82^, ^83^, ^84^ ^]^

To further understand the biochemical mechanism of glycine oxidase with respect to glyphosate, a fractional activity study was performed. Glyphosate is considered a competitive inhibitor as it competes against glycine for binding sites on the enzyme. The fractional activity at each inhibitor concentration was obtained and represents the glycine oxidase activity with regard to glycine in the presence of glyphosate (Figure 4c). In short, the activity of glycine oxidase was measured with its correct substrate, glycine, and a decrease in fractional activity was observed at increasing concentrations of glyphosate as glyphosate led to a decrease in the normal enzymatic binding of glycine. The fractional activity versus inhibitor concentration yielded an IC_50_ value of 12.16 mm and was estimated using a previously described methodology;^[^ ^85^ ^]^ this is the amount of glyphosate required to inhibit the glycine oxidase activity by 50% (i.e., where the fractional activity = 0.5). Since glyphosate is a known competitive inhibitor of glycine oxidase,^[^ ^83^ ^]^ the IC_50_ value could then be converted to a *K*_i_ value utilizing reported calculation methods for competitive inhibition (*K*_i_ = 1.69 mm).

### 2.3 Glyphosate Sensor Fabrication and Tuning

**Figure 5:**
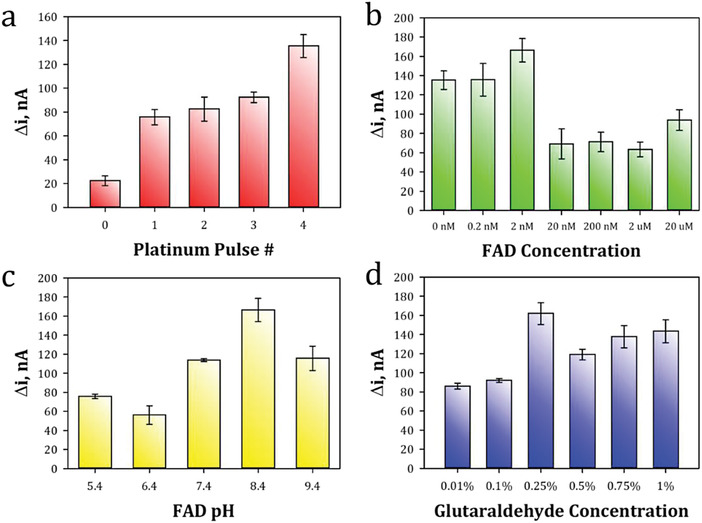
Sensor tuning studies and response (*n* = 3) to 100 µm glyphosate: a) increasing Pt pulses from 0 to 4 pulses, b) increasing flavin adenine dinucleotide (FAD) concentrations from 0 nm to 20 µm, c) increasing FAD pH values from 5.4 to 9.4, and d) increasing glutaraldehyde concentrations from 0.01% to 1%.

The sensor platform incorporates various elements that improve the response to glyphosate. To begin, a conductive platform is first created through the laser irradiation of the polyimide film. As is the case with most oxidase enzymes, catalytic nanoparticles are essential for the turnover of certain molecules into an electronic signal. Therefore, PtNPs were deposited and further studied to tune the sensor. Lastly, enzyme activity and attachment were studied regarding the presence of a cofactor and crosslinking agent. The elements directly studied included Pt pulses during electrodeposition, FAD concentration, FAD pH, and glutaraldehyde concentration (**Figure** **5**a–d). Increasing Pt pulses not only increased the ESA as shown in Table S2, Supporting Information, but allowed for more oxidation sites. As mentioned previously, glyphosate is oxidized by glycine oxidase and produces hydrogen peroxide. With increasing Pt pulses, more sites were available to turn over hydrogen peroxide into a readable signal. We opine that a point is reached when there is too much Pt deposited, more and more nanoparticles turn into micron sized features that decrease the enhanced catalytic effect of nanoparticles, the ESA for heterogeneous charge transport, and enzyme functionalization, and limits the ability for enhanced mass transport via radial diffusion.^[^ ^86^ ^]^ Consequently, 4 pulses were chosen as the CV current peaks converged between 3 and 4 pulses (Figure 3a), and the greatest response to glyphosate was observed at 4 pulses (Figure 5a).

To further probe methods to improve the performance of the Pt‐GlyOx‐LIG sensor, FAD concentration and pH were varied (Figure 5b,c). FAD is covalently linked to a dinucleotide with an adenine base. This molecule acts as a cofactor with glycine oxidase because of its redox capabilities.^[^ ^87^ ^]^ Cofactors aid in the stabilization of transition states, donate and/or accept electrons, as well as form key intermediates in the production process of biochemical reactions. In the glycine oxidase reaction mechanism, the oxidized form of FAD acts as an electron acceptor and is reduced to FADH_2_. Though there was not a strong dependence on the FAD concentration compared to the absence of FAD, a noticeable increase in signal was observed at 2 nm FAD. Furthermore, the pH of FAD was varied from 5.4–9.4. It is apparent that the greatest response was observed at a pH of 8.4. The isoelectric point of enzymes is a measure of the pH when a molecule containing both acidic and alkaline functional groups can be observed to have a neutral charge. The isoelectric point of GlyOx was determined to be at a pH of 5.8 ± 0.2.^[^ ^84^ ^]^ We hypothesize that the increased pH value in Figure 5c causes the enzyme active site to be in the reduced form, which made the active site more reactive and thus caused deamination to occur at a greater rate.

Furthermore, glutaraldehyde was used to immobilize the FAD‐GlyOx solution onto LIG as the aldehydes in glutaraldehyde will react with the hydroxyl groups on the LIG and amine groups on the enzyme.^[^ ^88^ ^]^ The concentration of glutaraldehyde was varied from 0.01–1% and showed a maximum response to glyphosate at 0.25% (Figure 5d). We hypothesize that as the glutaraldehyde concentration increased from 0.01%, more enzyme was crosslinked to the electrode surface. However, concentration increases after 0.25% showed a decrease in amperometric response presumably as more glutaraldehyde‐to‐enzyme crosslinking occurred, which effectively blocked more enzyme active sites and consequently hindered the enzyme GlyOx from reacting with its substrate, glyphosate. Note that the fabrication time for a batch of 10 fully functionalized sensors takes roughly 32 min and these can be tested the following day.

### 2.4 Electrochemical Sensing of Glyphosate

**Figure 6:**
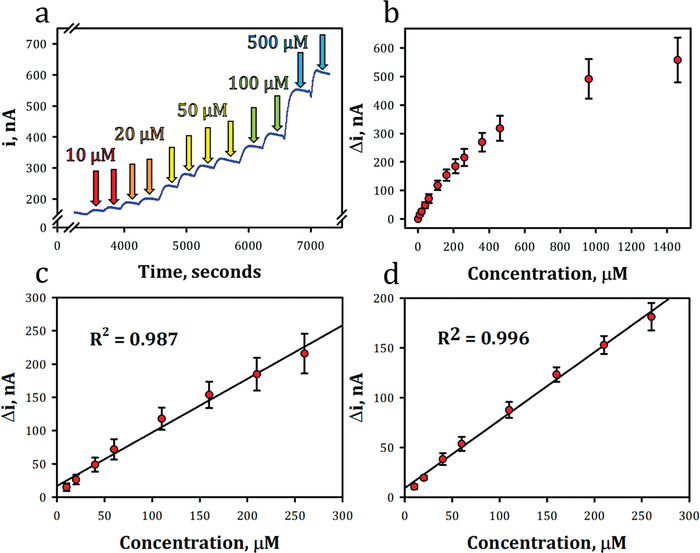
Electrochemical detection of glyphosate: a) current versus time plot of glyphosate additions, b) sensitivity plot of glyphosate for *n* = 10 sensors, c) linear range of glyphosate detection for *n* = 10 sensors, and d) linear range of glyphosate detection for *n* = 3 repeats on a single sensor.

Glyphosate was detected amperometrically at a constant potential of +0.6 V versus Ag/AgCl in PBS pH 7.4. Glyphosate, a glycine‐like derivative and synthetic amino acid, is oxidized by glycine oxidase which produces hydrogen peroxide, glyoxylate and ammonia. Once hydrogen peroxide is produced, the molecule can be oxidized at +0.6 V versus Ag/AgCl, and the decomposition of hydrogen peroxide is further catalyzed by PtNPs.^[^ ^89^ ^]^ Through this oxidation, two electrons are released and read as an amperometric signal. Therefore, the concentration of glyphosate can be directly correlated to a change in current. **Figure** **6**a plots the amperometric response against increasing concentrations of glyphosate. Responses appear in a stepwise manner, and the signal plateaued as the enzymatic reaction became saturated with glyphosate at concentrations over 1400 µm. The glyphosate Pt‐GlyOx‐LIG sensor exhibited a response time of 150 s. Figure 6b,c displays the sensitivity of the sensors to glyphosate for *n* = 10 sensors with a relative standard error ranging from 3.9–11.8% and a coefficient of determination of 0.987. The sensors demonstrated a linear range from 10–260 µm, sensitivity of 0.991 nA µm ^−1^ and LOD of 3.03 µm, which was calculated using the 3‐sigma method.^[^ ^90^ ^]^ Furthermore, to test the variability of a singular sensor over multiple uses, repeats were performed (*n* = 3) and displayed a relative standard error ranging from 3.4–13.6% and a determination coefficient of 0.996 (Figure 6d), highlighting the ability to reuse the Pt‐GlyOx‐LIG sensor.

**Figure 7:**
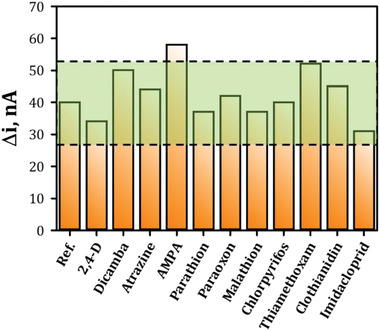
Interferent study. Investigation of common herbicides, organophosphate insecticides, and neonicotinoids with a green box encompassing 1 standard deviation above and below the glyphosate reference response to illustrate the expected variability of a glyphosate sensor in the absence of interference. Bars represent the amperometric response to 50 µm glyphosate in the presence of the potential interferent species.

Additionally, the Pt‐GlyOx‐LIG sensor was tested against various interferent pesticides, including herbicides: atrazine, dicamba and 2,4‐dichlorophenoxyacetic acid (2,4‐D); organophosphate insecticides: parathion‐methyl, paraoxon‐methyl, malathion and chlorpyrifos; neonicotinoids: imidacloprid, clothianidin and thiamethoxam; and the glyphosate degradation product AMPA. Results are plotted in **Figure** **7**, and chemical structures of all pesticides can be found in Figure S3, Supporting Information. Interferent pesticides were tested in the presence of glyphosate to observe if there was a change in the expected signal to glyphosate. First, 50 µm of the interferent species was added followed by 50 µm of glyphosate. Potential interferent species were deemed non‐interferents if the response to glyphosate remained within ±1 standard deviation of the expected signal to glyphosate based upon the calibrated model. This indicates that the change in signal more than likely reflects variability among a batch of sensors. The green box represents 1 standard deviation (13 nA) above and below the glyphosate reference. This standard deviation was taken as the average standard deviation from the calibrated model at 50 µm. Herbicides 2,4‐D, dicamba, and atrazine showed negligible interference. Similarly, the organophosphate insecticides: parathion‐methyl, paraoxon‐methyl, malathion and chlorpyrifos as well as neonicotinoids: thiamethoxam, clothianidin and imidacloprid showed negligible interference. In contrast, AMPA showed an increased response, which is attributed to the signal obtained when the enzyme functions under the AMPA pathway.

To further demonstrate the capabilities of the Pt‐GlyOx‐LIG sensor, recovery tests were performed in complex fluids, including river water from the South Skunk River in Iowa and crop residues. See the Experimental Section for river water and crop residue preparation. Recovery responses are reported in **Table** **1**. For *n* = 3 responses to 50 µm glyphosate, the average recovery percentages in river water, corn residue and soybean residue were 92.5%, 109.1% and 124.9%, respectively. River water data exhibited a slightly reduced signal, which could be due to other organic and inorganic species that may foul the surface of the electrode, interact with the enzyme or obstruct charge transfer. Corn and soybean residue data exhibited slightly higher recoveries, which is attributed to the oxidation of the innate glycine composition in each crop. These results confirm the viability of applying the Pt‐GlyOx‐LIG sensor in‐field applications. It is noted that the 3.03 µm LOD of this sensor ensures the detection of the EPA's maximum contaminant level for glyphosate at 0.7 mg L^−1^ (4.1 µm).^[^ ^91^ ^]^

**Table 1:** Complex fluid recovery test

Complex fluid	Average measured concentration [µm]	Recovery percentage [%]
River water	46.3 ± 6.9	92.5
Corn	54.6 ± 2.9	109.1
Soybeans	62.4 ± 8.7	124.9

## 3 Conclusions

In conclusion, this work demonstrates the first use of glycine oxidase as well as a laser‐induced graphene platform for the selective detection of glyphosate. The rapid production of Pt‐decorated LIG sensors showcases the high‐throughput and scalability of this fabrication method, which eliminates rigorous graphene synthesis and exfoliation methods, thermal annealing and ink formulation. Additionally, LIG possesses remarkable electrical properties, electrocatalytic sites, large electrochemical surface area, and rich functional groups that are conducive to biosensing. The Pt‐GlyOx‐LIG sensor displayed a linear range of 10–260 µm, LOD of 3.03 µm, and response time of 150 s. Additionally, this sensor explored the widest range of interferent pesticides of any glyphosate biosensor and confirmed that glycine oxidase shows minimal interference from atrazine, 2,4‐D, dicamba, parathion‐methyl, paraoxon‐methyl, malathion, chlorpyrifos, thiamethoxam, clothianidin, and imidacloprid. Furthermore, the sensor accurately reported glyphosate concentrations in river water, corn residue and soybean residue with recovery percentages of 92.5%, 109.1% and 124.9%, respectively.

The scope of this work generates broader implications concerned with point‐of‐use sensing and environmental monitoring. A user‐friendly, electrochemical pesticide sensor enables agricultural stakeholders to further map potential pesticide migration from the point of application to unintended surface and groundwaters and additionally promotes smart food packaging through crop residue analysis. The demonstrated one‐step lasing process displays a rapid manufacturing procedure that can be incorporated into various fields that require a conductive and high surface area platform, which may include energy and battery applications, health monitoring devices and strain sensors. The investigation and incorporation of redox mediators to avoid Pt consumption, surface treatments to improve the electroactive behavior of the LIG, and pumpless microfluidics to simplify the electrochemical cell constitute future work that could be performed to improve the ease of fabrication, robustness and sensitivity of the sensor.

## 4 Experimental Section

### Materials and Reagents

Phosphate buffer saline with potassium chloride (10 mm) tablets at pH 7.4, glutaraldehyde (25%), flavin adenine dinucleotide, sodium chloride, perchloric acid (70%), chloroplatinic acid (8%), glyphosate, atrazine, aminomethylphosphonic acid, thiamethoxam, imidacloprid, clothianidin, paraoxon‐methyl, parathion‐methyl, malathion and chlorpyrifos were purchased from Sigma Aldrich. 2,4‐dichlorophenoxyacetic acid was purchased from Tokyo Chemical Industry, and dicamba was provided by the Iowa State University chem service. The glycine oxidase gene originates from *Bacillus subtilis* and gives rise to a 47 kDa protein.^[^ ^84^, ^92^ ^]^ Glycine oxidase (40 µm) was prepared by the U.S. Naval Research Laboratory in a manner similar to that described in references.^[^ ^93^, ^94^ ^]^

Glutaraldehyde dilutions were prepared in PBS pH 7.4. Flavin adenine dinucleotide dilutions were prepared in PBS in a pH range from 5.4–9.4. All pesticides were prepared in 10× PBS pH 7.4. Kapton polyimide substrate (0.125 µm thick) was purchased from McMaster‐Carr. River water samples were gathered from the South Skunk River in Iowa. Crop residues were tested on crops purchased from a local grocery market.

### Pt‐LIG Fabrication and Characterization

Kapton polyimide film was taped to an aluminum plate, washed with isopropyl alcohol, and placed on the bed of a 75 W Epilog Fusion M2 CO_2_ laser. The laser was defocused 2 mm and operated at 7% speed, 7% power, 50% frequency, and 1200 dots per inch. The electrode design was prepared in CorelDRAW. The laser converted the polyimide film into a 3 mm diameter LIG dipstick. Acrylic polish was applied over the stem of the electrode to act as a passivation layer and maintain a constant working area. Using a 3‐electrode cell with a platinum wire counter, Ag/AgCl reference (0.1 m KCl), and LIG working electrode, a potential step function was applied in perchloric acid (0.1 m) and chloroplatinic acid (5 mm) over 30 s at −0.5 V to deposit platinum nanoparticles on the LIG surface. Platinum pulses were varied 0 to 4. SEMs were taken with an FEI Quanta 250 FEG scanning electron microscope at a 10 kV accelerating voltage. The displayed SEMs are secondary electron images. XPS measurements were performed using an Amicus from Kratos Analytical. Raman measurements were performed using a Horiba XploRA Plus confocal Raman microscope equipped with a 532 nm laser operating at 1.2 mW and a 50× objective (0.5 NA). 12 Raman spectra were collected at 12 randomly selected locations and each Raman spectrum were collected with a 30 s acquisition and 3 accumulations. All Raman peaks in each spectrum were fitted to a Lorentzian function in Igor Pro 6.37 to calculate the average *I*_2D_/*I*_G_ ratio.

### Enzyme Solution Preparation

Glycine oxidase, glutaraldehyde, and FAD were mixed in an equal volumetric ratio. The solution was drop coated onto a petri dish and repeatedly pipetted and discharged 20 cycles from the tip to further mix the solution. 3 µL of the solution was pipetted onto the working portion of each electrode. After 2 min, the pipetted solution was removed so as to leave a thin film that wetted the surface of the LIG. Sensors were stored at 8 °C overnight and were not removed until tested.

### Michaelis–Menten Assay of Glycine Oxidase

A 200 nm glycine oxidase stock solution was made using 1× assay buffer provided in the glycine assay kit (Cell Biolabs, MET‐5070) and kept on ice. To a 384‐well plate, 5 µL of stock enzyme solution was added followed by 5 µL of the master reaction mix, which was composed of 0.1 µL fluorescent probe per well, 0.02 µL per well horseradish peroxidase and 4.88 µL per well 1× buffer. All of which were provided by the kit. The plate was spun briefly at 300 rpm to ensure all droplets were at the bottom of the well. A 2 m glycine stock solution was made with 1× assay buffer. This was diluted serially to achieve glycine stocks ranging in concentration from 49 to 0.05 mm. To start the reaction, 10 µL of glycine at each concentration was added to the 384‐well plate containing enzyme. Each glycine concentration measurement was performed in triplicate. The plate was immediately covered with a piece of film and placed in a Tecan Spark dual monochromator multifunction plate reader where a kinetic program was started. The kinetic program consisted of shaking the plate for 10 s to ensure adequate mixing, the excitation was set to 500 nm (20 nm bandwidth), and the emission wavelength was set to 595 nm (5 nm bandwidth). The emission was recorded at 595 nm every 25 s for up to 2 h at 37 °C with the gain manually set to 85. The data was analyzed by constructing a calibration curve using the last time points of the progress curves plotted against the glyoxylate concentration which is assumed to be proportional to the glycine concentration according to the kit. To determine the kinetic characteristics of glycine oxidase, the initial rates for each concentration of glycine was determined by calculating the slopes of the linear portions of the progress curves. These initial rates where plotted against glycine concentration and fitted to the Michaelis–Menten equation by minimizing the error between the estimated initial rate and the actual initial rate when solving for *K*_M_ and *V*_max_.

### Inhibition Assay with Glyphosate

A 200 mm stock solution of glycine was made using the 1× PBS assay buffer provided in the glycine assay kit which was aliquoted in equal concentrations across variable glyphosate solutions prepared from a 50 mm stock. The final concentrations of the components were as follows: 12 mm glycine and 0–22 mm glyphosate. Using the glycine fluorescence‐based assay kit (Cell Biolabs, MET‐5070), the excitation was set to 550 nm (20 nm bandwidth), and the emission wavelength was set to 595 nm (5 nm bandwidth). The emission was immediately measured at 595 nm using a Tecan Spark dual monochromator multifunction plate reader and a kinetic program consisting of shaking the plate for 10 s before measuring the emission at 37 °C for 2 h with the gain manually set to 85. The emission values were converted to glyoxylate concentrations using the standard curve prepared from the Michaelis–Menten data. Pipetting was performed using standard pipettes.

All activity measurements were performed as three replicates. The linear portions of the progress curves were used to calculate the initial rates for each inhibitor concentration. The fractional activity at each inhibitor concentration was obtained as the ratio of the initial rate at 12 mm glycine in the presence of the inhibitor (glyphosate) to the initial rate at 12 mm glycine in absence of the inhibitor (glyphosate) at each inhibitor concentration. From this, an IC_50_ value was extrapolated.

### Electrochemical Analysis

A CH Instruments potentiostat was used for all electrochemical measurements. For the 3‐electrode setup, a commercial platinum counter wire and Ag/AgCl reference (0.1 m KCl) were purchased from CH instruments. The electrode system was submerged in 10 mL of the 10× PBS pH 7.4 solution with an applied potential of +0.6 V at a stir rate of 120 rpm. Note that the PBS was not deoxygenated. Amperometry was used to record the current from the glyphosate oxidation. Results were reported as the change in current between the plateau of each glyphosate addition and the recorded baseline before the additions. Recovery percentages in river water and crop residues were calculated with respect to the spiked concentration in the complex fluid and the measured concentration from the calibrated model given the amperometric response in the complex fluid.

### Interference Studies

Interferent pesticides were tested by observing the change in current after a 50 µm interferent pesticide addition that was followed by a 50 µm glyphosate addition. The change in current was reported as the difference between the stabilized signal prior to the interferent pesticide addition and the response to glyphosate. The reference bar is the change in current to 50 µm of glyphosate in the absence of potential interferent pesticides.

### Complex Fluid Sample Preparation

River water samples were taken from the South Skunk River near Ames, Iowa. River samples were filtered through a 3.1 µm glass fiber filter and brought to a concentration of 0.5 m NaCl as a supporting electrolyte. Electrochemical analysis was performed in 10 mL of this river water solution. Corn and soybeans were purchased from a local grocery store. Corn kernels were removed from the husk and mashed in a mortar and pestle. Soybeans were blended. Mash slurry was prepared by measuring 3 g of either the corn or soybean mash and was mixed in 12 mL of 10× PBS pH 7.4. Samples were centrifuged at 4500 rpm for 15 min. Supernatant liquid was removed and brought to 10 mm glyphosate. This spiked residue solution was used for electrochemical analysis.

### Statistical Analysis

All data was recorded using a CHI potentiostat and saved as a CSV file. Values were reported as the mean ± standard deviation. For sensor tuning studies, multiple glyphosate sensors, and repeats on a single sensor, experiments were performed with *n* = 3, *n* = 10 and *n* = 3 samples, respectively.

## Conflict of Interest

The authors declare no conflict of interest.

## Supporting information

Supporting information

## Data Availability

The data that support the findings of this study are available from the corresponding author upon reasonable request.
