# Supplementary material for: Enzymatic Laser‐Induced Graphene Biosensor for Electrochemical Sensing of the Herbicide Glyphosate
Source: Glob Chall. 2022 Jul 26;6(9):2200057. doi: 10.1002/gch2.202200057 (PMC9463521; doi:10.1002/gch2.202200057)
Supplement: Supplementary file 1 — Supporting information [file GCH2-6-2200057-s001.pdf]

Global Challenges
Open Access

Supporting Information

for *Global Challenges,* DOI: 10.1002/gch2.202200057

Enzymatic Laser-Induced Graphene Biosensor for Electrochemical Sensing of the Herbicide Glyphosate

*Zachary T. Johnson, Nathan Jared, John K. Peterson, Jingzhe Li, Emily A. Smith, Scott A. Walper, Shelby L. Hooe, Joyce C. Breger, Igor L. Medintz, Carmen Gomes, and Jonathan C. Claussen**

Selective Detection of Glyphosate Using Glycine Oxidase on Platinum-decorated Laser-Induced Graphene

Zachary T. Johnson, Nathan Jared, John K. Peterson, Jingzhe Li, Emily A. Smith, Scott A. Walper, Shelby L. Hooe, Joyce C. Breger, Igor L. Medintz, Carmen Gomes, and Jonathan C. Claussen[*]

Supporting Information

Electroactive Surface Area Values for Calculations

D = 7.6 E-6 cm^2 s^{-1}

n = 1 electron

C = 5 mM = 0.000005 mol cm^{-3}

v = scan rate

i_p = peak current

Table S1. Electroactive Surface Area for Varying Platinum Depositions.

**Platinum Pulse #**	0	1	2	3	4
**ESA, cm^2**	0.127	0.165	0.169	0.186	0.193
**Percent of Geometric Surface Area, %**	179	233	238	262	272

Transfer Coefficient

Cyclic voltammogram data can be collected over varying scan rates. To calculate the transfer coefficient, α, the current *vs.* potential data must be gathered along the decreasing portion of the cathodic peak. The log(current) *vs.* the potential is plotted for this region and the slope, m, at each scan rate can be found and denoted as the Tafel slope. Using **equation S1**, α can be calculated and averaged over each scan rate, where R is the gas constant, T is the absolute temperature, and F is Faraday's constant.

$$\alpha = -\frac{2.3\mathrm{mRT}}{\mathrm{F}} \qquad (1)$$

Nicholson Method for the Calculating Heterogeneous Electron Transfer Rate

From the cyclic voltammagram data, ΔE_p may be found for each scan rate. **Equation S2** is derived from the data shown in **Table S2.**[1] This approach relates the peak to peak separation, ΔE_p, with the dimensionless Nicholson parameter, Λ as described by Aristov[2] or Ψ as described by Nayak[1] and Muhammad.[3] Furthermore, Ψ is then related to the heterogeneous electron transfer rate, k^0, by **equation S3**. Electrochemical reversibility is determined from **Table S3**.

$$\Psi = \frac{-0.6288 + 0.0021\ \Delta E_p}{1 - 0.017\ \Delta E_p} \tag{2}$$

Table S2. Relationship between Nicholson Parameter, Λ, and ΔE_p (reference from Table 2[2])

**ΔE_p, mV**	60	64	66	68	70	90	100	110	120	130	160
**Λ**	19	5.1	3.63	2.81	2.26	0.77	0.57	0.44	0.36	0.29	0.19

$$k^0 = \Psi \sqrt{\frac{\pi DnFv}{RT}} \tag{3}$$

Table S3. Electrochemical Reversibility Criteria (reference from Table 3[2])

**Reversible**	$\Lambda > 10$	$k_0 > 0.35\ v^{1/2}$
**Quasi-reversible**	$10 > \Lambda > 10^{-2}$	$0.35\ v^{1/2} > k_0 > 3.5(10^{-4})\ v^{1/2}$
**Irreversible**	$\Lambda < 10^{-2}$	$k_0 < 3.5(10^{-4})\ v^{1/2}$

Kochi Method for the Calculating Heterogeneous Electron Transfer Rate

The Kochi method calculates the electron transfer rate based upon the transfer coefficient, α, which was determined using the Tafel slope method, and the peak to peak separation, ΔE_p. The rate is determined using **equation S4**.

$$k^0 = 2.18 \sqrt{\frac{\alpha DnFv}{RT}} \exp\left[\frac{-\alpha^2 nF(\Delta E_p)}{RT}\right] \tag{4}$$

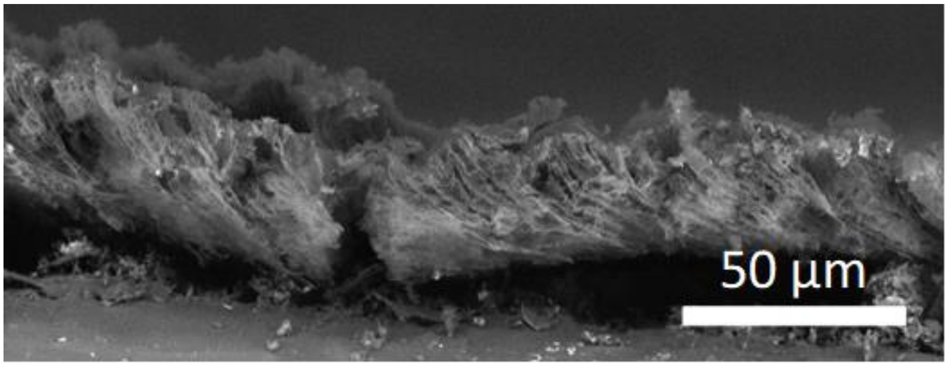

Figure S1. SEM cross sectional image of LIG.

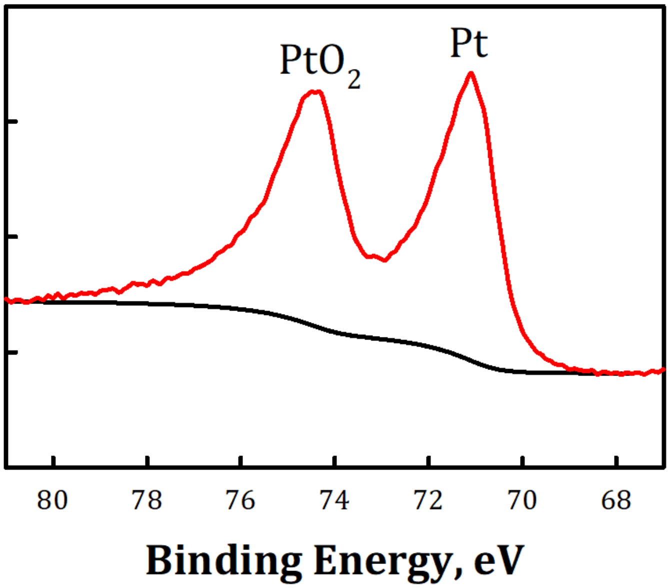

Figure S2. XPS of Pt 4f region showing distinct metallic Pt and PtO_2.

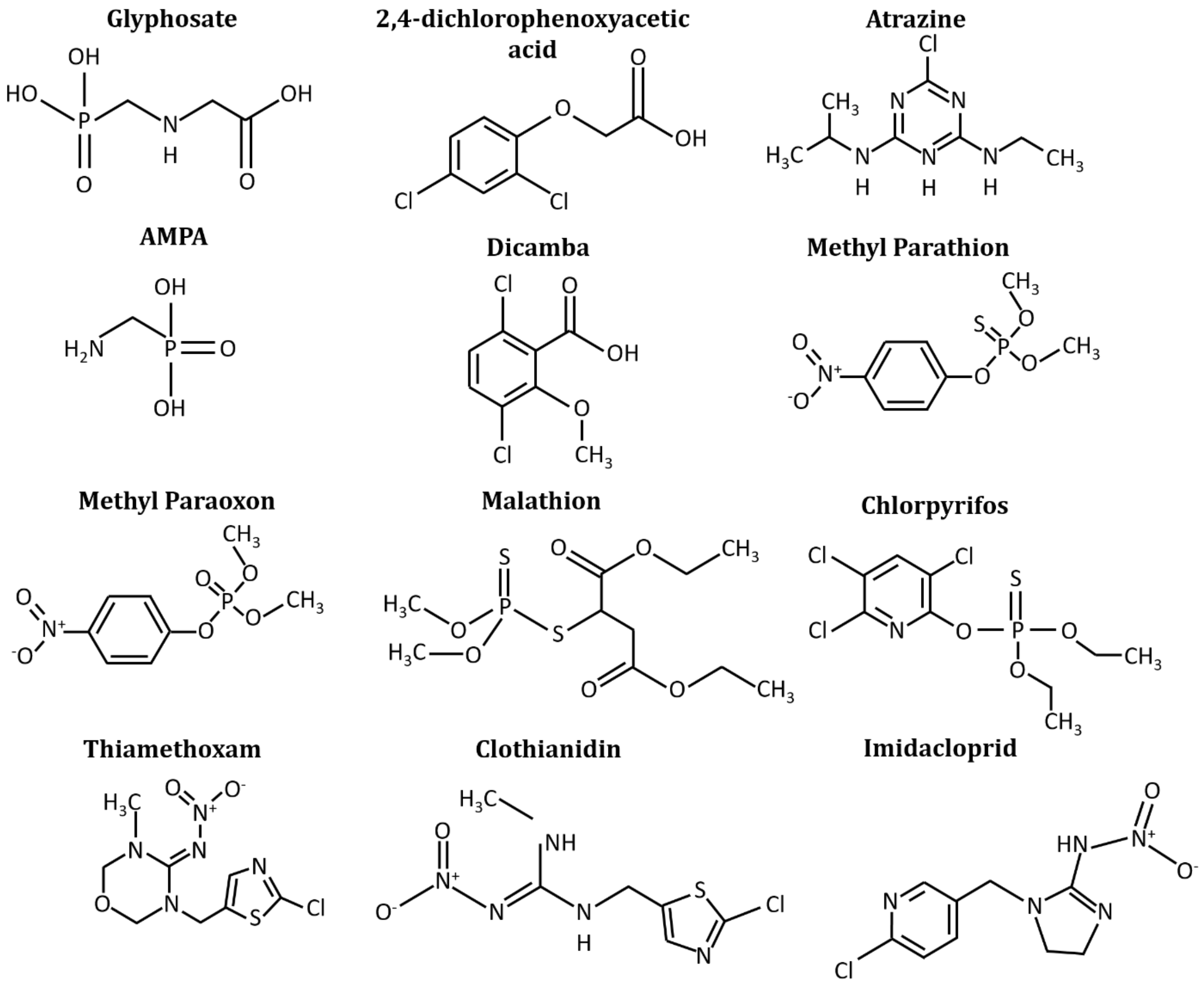

Figure S3. Chemical structures of referenced herbicides, insecticides, and degradation products tested throughout this study.

References

[1] P. Nayak, N. Kurra, C. Xia, H. N. Alshareef, *Adv. Electron. Mater.* **2016**, *2*, 1600185.

[2] N. Aristov, A. Habekost, *World J. Chem. Educ.* **2015**, *3*, 115.

[3] H. Muhammad, I. A. Tahiri, M. Muhammad, Z. Masood, M. A. Versiani, O. Khaliq, M. Latif, M. Hanif, *J. Electroanal. Chem.* **2016**, *775*, 157.
